# Supplementary material for: Overexpression of DGAT2 Stimulates Lipid Droplet Formation and Triacylglycerol Accumulation in Bovine Satellite Cells
Source: Animals (Basel). 2022 Jul 20;12(14):1847. doi: 10.3390/ani12141847 (PMC9312262; doi:10.3390/ani12141847)
Supplement: Supplementary file 1 [file animals-12-01847-s001.zip › animals-1742011-supplementary.pdf]

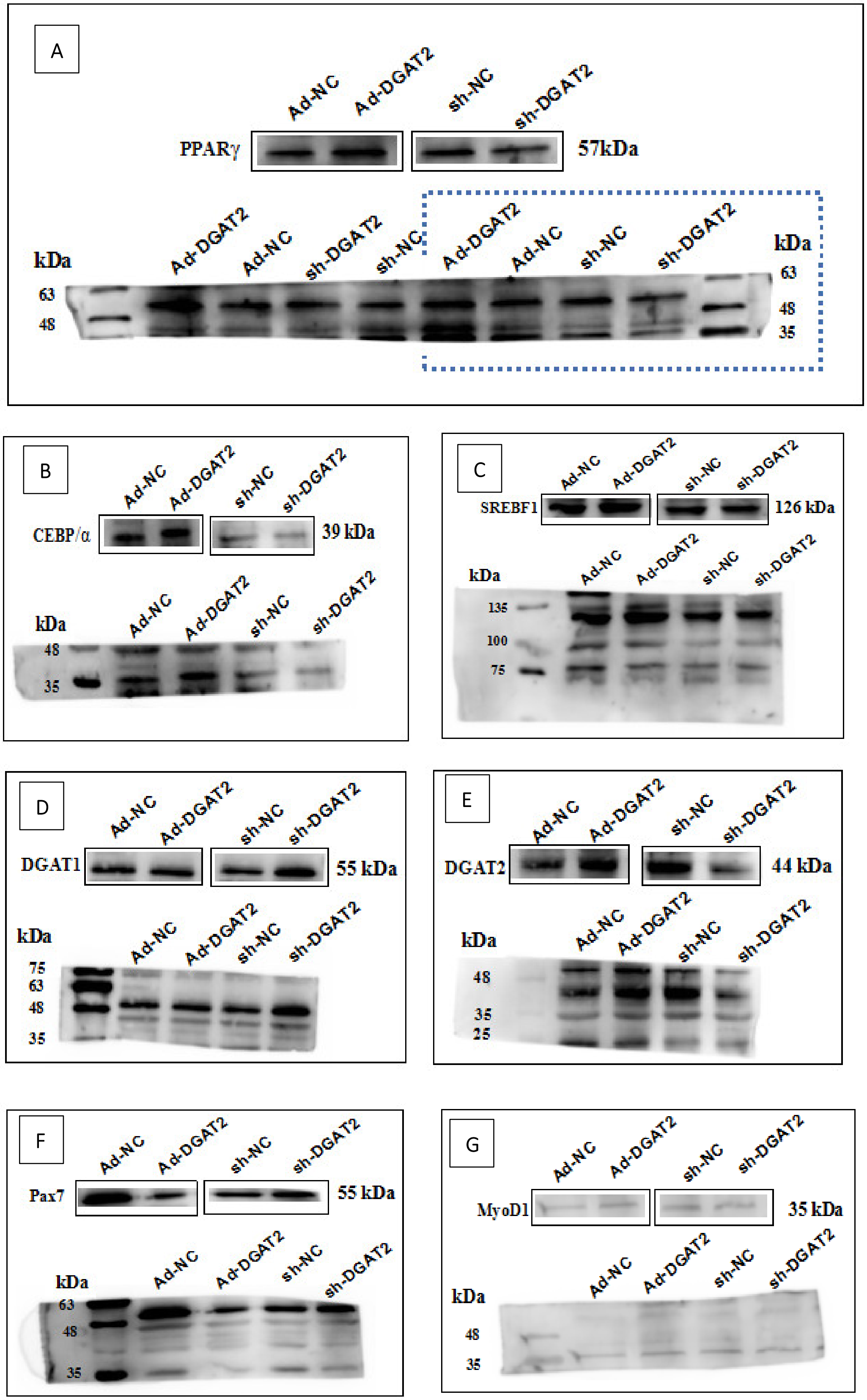
A
Ad-NC
Ad-DGAT2
sh-NC
sh-DGAT2
PPARγ
57kDa
kDa
63
48
35
B
CEBP/α
39 kDa
C
SREBF1
126 kDa
135
100
75
D
DGAT1
55 kDa
E
DGAT2
44 kDa
25
F
Pax7
55 kDa
G
MyoD1
35 kDa

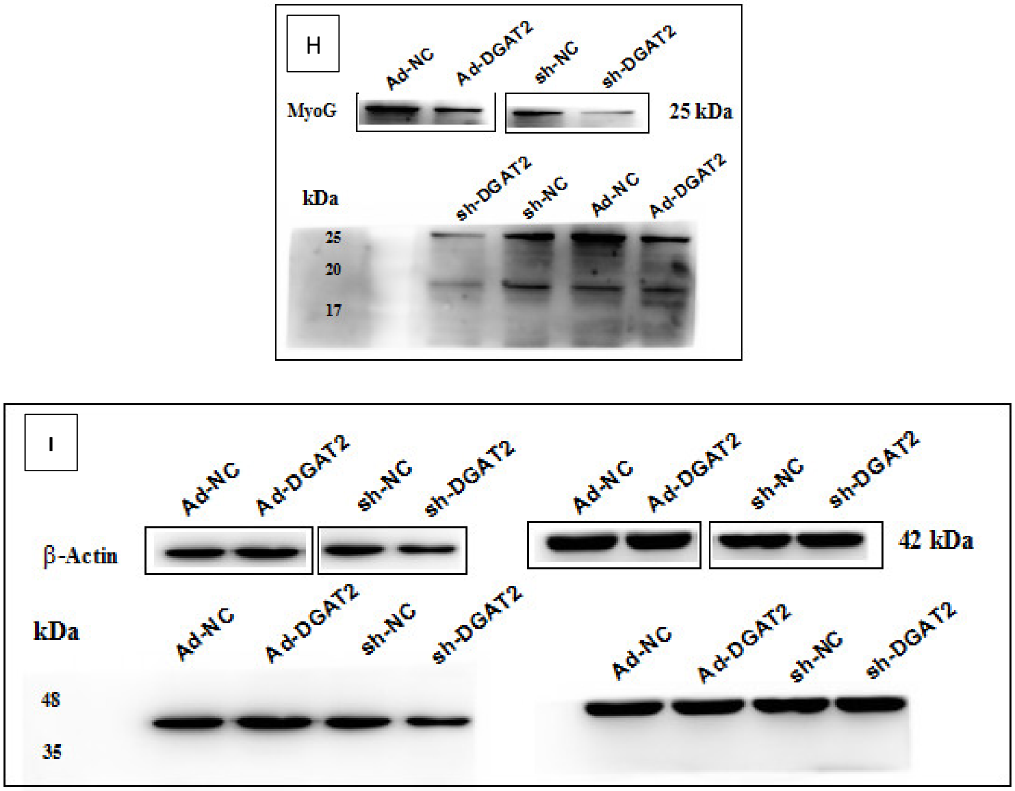


**Figure S1.** The original figure of the upper blot membrane of Figure 5E and 6E.The original western blot of PPARγ (**A**), C/EBPα (**B**), SREBF1 (**C**), DGAT1 (**D**), DGAT2 (**E**), Pax7 (**F**), MyoD1 (**G**), MyoG (**H**) and β-actin (**I**) protein in BSCs transfected with Ad-DGAT2/Ad-NC and sh-DGAT2/sh-NC.

**Table S1.** Information of clean data.

| Sample | Trimmed_Read_Number | Trimmed_Bases | Useful_Read% | Useful_Bases% |
|---|---|---|---|---|
| Ad-DGAT2-1 | 39262616 | 5.89E+09 | 93.31 | 93.31 |
| Ad-DGAT2-2 | 35167218 | 5.28E+09 | 93.21 | 93.21 |
| Ad-DGAT2-3 | 38882100 | 5.83E+09 | 93.13 | 93.13 |
| Ad-NC-1 | 46321320 | 6.95E+09 | 92.71 | 92.71 |
| Ad-NC-2 | 42544930 | 6.38E+09 | 92.75 | 92.75 |
| Ad-NC-3 | 40137942 | 6.02E+09 | 92.9 | 92.9 |
| sh-NC-1 | 39417084 | 5.91E+09 | 93.48 | 93.48 |
| sh-NC-2 | 36545664 | 5.48E+09 | 92.79 | 92.79 |
| sh-NC-3 | 40611026 | 6.09E+09 | 92.55 | 92.55 |
| sh-DGAT2-1 | 35169252 | 5.28E+09 | 92.93 | 92.93 |
| sh-DGAT2-2 | 35238180 | 5.29E+09 | 92.85 | 92.85 |
| sh-DGAT2-3 | 38945646 | 5.84E+09 | 91.95 | 91.95 |

**Table S2.** Summary of sequencing dada and reference genome compar-ison.

| Sample | Clean Reads | Total Mapped | Multiple Mapped | Uniquely Mapped | Map Events | Mapped to Gene | Mapped to InterGene | Mapped to Exon |
|---|---|---|---|---|---|---|---|---|
| Ad-DGAT2-1 | 39262616 | 37622923 (95.82%) | 873691 (2.32%) | 36749232 (97.68%) | 36749232 | 35293712 (96.04%) | 1455520 (3.96%) | 31542318 (89.37%) |
| Ad-DGAT2-2 | 35167218 | 33651647 (95.69%) | 786025 (2.34%) | 32865622 (97.66%) | 32865622 | 31549754 (96.00%) | 1315868 (4.00%) | 28197356 (89.37%) |
| Ad-DGAT2-3 | 38882100 | 37271804 (95.86%) | 871345 (2.34%) | 36400459 (97.66%) | 36400459 | 34913834 (95.92%) | 1486625 (4.08%) | 31094804 (89.06%) |
| Ad-NC-1 | 46321320 | 44601943 (96.29%) | 1063563 (2.38%) | 43538380 (97.62%) | 43538380 | 41855359 (96.13%) | 1683021 (3.87%) | 37777213 (90.26%) |
| Ad-NC-2 | 42544930 | 40999061 (96.37%) | 998055 (2.43%) | 40001006 (97.57%) | 40001006 | 38455274 (96.14%) | 1545732 (3.86%) | 34845919 (90.61%) |
| Ad-NC-3 | 40137942 | 38686409 (96.38%) | 916427 (2.37%) | 37769982 (97.63%) | 37769982 | 36307118 (96.13%) | 1462864 (3.87%) | 32804176 (90.35%) |
| sh-NC-1 | 39417084 | 37878441 (96.10%) | 910491 (2.40%) | 36967950 (97.60%) | 36967950 | 35564990 (96.20%) | 1402960 (3.80%) | 32005234 (89.99%) |
| sh-NC-2 | 36545664 | 35113371 (96.08%) | 821878 (2.34%) | 34291493 (97.66%) | 34291493 | 32980521 (96.18%) | 1310972 (3.82%) | 29687377 (90.01%) |
| sh-NC-3 | 40611026 | 39064222 (96.19%) | 941925 (2.41%) | 38122297 (97.59%) | 38122297 | 36658899 (96.16%) | 1463398 (3.84%) | 33056041 (90.17%) |
| sh-DGAT2-1 | 35169252 | 33751703 (95.97%) | 819747 (2.43%) | 32931956 (97.57%) | 32931956 | 31668648 (96.16%) | 1263308 (3.84%) | 28467607 (89.89%) |
| sh-DGAT2-2 | 35238180 | 33889997 (96.17%) | 823194 (2.43%) | 33066803 (97.57%) | 33066803 | 31823353 (96.24%) | 1243450 (3.76%) | 28581042 (89.81%) |
| sh-DGAT2-3 | 38945646 | 37444094 (96.14%) | 900685 (2.41%) | 36543409 (97.59%) | 36543409 | 35145388 (96.17%) | 1398021 (3.83%) | 31473172 (89.55%) |
